# Vaginal microbiomes of breast cancer survivors treated with aromatase inhibitors with and without vulvovaginal symptoms

**DOI:** 10.1038/s41598-024-58118-3

**Published:** 2024-03-28

**Authors:** Pimpun Prasanchit, Pisut Pongchaikul, Panuwat Lertsittichai, Chananya Tantitham, Jittima Manonai

**Affiliations:** 1 Department of Obstetrics & Gynaecology, Faculty of Medicine Ramathibodi Hospital, Mahidol University, 270, Rama VI Rd., Bangkok, 10400 Thailand; 2 Ramathibodi Medical School, Faculty of Medicine Ramathibodi Hospital, Mahidol University, 111, Suwannabhumi Canal Rd., Samut Prakan, 10540 Thailand; 3 Department of Surgery, Faculty of Medicine Ramathibodi Hospital, Mahidol University, 270, Rama VI Rd., Bangkok, 10400 Thailand; 4 Integrative Computational BioScience Center, Mahidol University, Nakhon Pathom, Thailand; 5 Institute of Infection, Veterinary and Ecological Sciences, University of Liverpool, Liverpool, United Kingdom

**Keywords:** Microbiology, Health care, Molecular medicine

## Abstract

Genitourinary syndrome of menopause (GSM) is the leading cause of vaginal symptoms in breast cancer survivors treated with aromatase inhibitors. However, there are currently no effective treatment options available for women with a history of breast cancer. Recent research has established that changes in the vaginal microbiome may be linked to GSM. Most studies have assessed the microbiome without accounting for the estrogen status. It remains unknown whether the vaginal microbiome differ among patients with a low estrogenic state with and without vulvovaginal symptoms. To address such research questions, our study compares the vaginal microbiomes among breast cancer survivors treated with aromatase inhibitors with and without vulvovaginal symptoms. A total of 50 breast cancer survivors treated with aromatase inhibitors were recruited, among whom 25 had vulvovaginal symptoms and 25 had no vulvovaginal symptoms. Vaginal swabs were collected. DNA extraction, followed by sequencing of the V3–V4 regions of the 16S ribosomal RNA gene, were performed. Differential abundance analysis was conducted by linear discriminant analysis effect size. Taxonomy assignment, alpha diversity and beta diversity were examined. The relative abundance of genus *Sneathia* and genus *Gardnerella* was significantly increased in vulvovaginal symptoms group with no differences in bacterial diversity and richness.

## Introduction

Breast cancer (BC) has been reported as the most commonly diagnosed cancer type globally in 2020 by the International Agency for Research on Cancer (IARC) with the increase in diagnosis rates showing a long-term upward trend that is predicted to continue^1^. Most patients with BC are postmenopausal, with the incidence and death rates generally being proportional to age until approximately the seventh decade^2^. By contrast, BC mortality rates have declined owing to improved and more targeted treatments and early detection through screening mammography. Currently, adjuvant medical therapy with aromatase inhibitors (AIs) for BC is considered appropriate for an initial duration of 5 years, according to the National Comprehensive Cancer Network (NCCN) Panel^3^. AIs work by directly inhibiting the aromatase enzyme responsible for the conversion of androgens to estrogens, resulting in a dramatic suppression of serum estrogen levels^4^. Patients treated with AIs show a significant decrease in serum estrone and estradiol compared with healthy postmenopausal women^5,6^. Genitourinary syndrome of menopause (GSM) associated with vulvovaginal atrophy (VVA) caused by low estradiol levels includes vaginal dryness, itching, discharge and dyspareunia, are also common in postmenopausal women^7,8^. VVA through local inhibition of aromatase is the leading cause of vaginal symptoms and sexual dysfunction in BC survivors (BCS) treated with AIs^7,9^. As a result of the low estradiol levels observed in healthy postmenopausal women with vaginal symptoms, local vaginal estradiol or estriol are commonly used for treatment in the form of tablets, creams, suppositories or rings^10^. Seeing as menopausal hormone therapy is not recommended in women with a history of BC because of possible disease recurrence, there are currently no effective treatment options available for VVA in BCS^9,10^.

Recent research has established a clear link between dysregulation of the microbiome and human disease. The microbiome is defined as a living ecosystem, which includes the entire population of microorganisms, their genomes and the surrounding environment^11^. The human female reproductive tract consists of a site-specific microbiome, the homeostasis of which is vital to health^12^. A disrupted microbial ecosystem or “dysbiosis” due to fluctuations in the growth rate and survival of each of its constituents might be associated with various vaginal health conditions, such as bacterial vaginosis, trichomonas vaginitis, sexually transmitted infections, aerobic vaginitis, and vulvovaginal candidiasis^12–16^. Multiple factors have been shown to affect the vaginal microbiome including the estrogen status. In postmenopausal women, changes in the vaginal microbiome may be linked to symptoms of VVA and dryness, which negatively affect their sexual health and quality of life^12,17^. *Lactobacillus* appears to play an important role in probiotic activity to maintain the vaginal ecosystem by contributing to the reinforcement of the host immune system against several primary and opportunistic pathogens, which is influenced not only by individual *Lactobacillus* species but also by their multi-microbial interaction as consortia^18–20^. Previous reports show a negative correlation between the ratio of *Lactobacillus* species and vaginal dryness, with greater bacterial diversity observed in those with moderate to severe dryness^21,22^. A predominance of *Lactobacillus* species in the vaginal microbiota has been linked with increased serum estrone levels^23^. Although it is acknowledged that the vaginal microbiome is essential for good vaginal health, most studies have assessed the constituents of the microbiome without accounting for the estrogen status or vaginal health. It remains unknown whether the components of the vaginal microbiome differ among patients with a low estrogenic state with and without vulvovaginal symptoms. A comprehensive understanding of the vaginal microbiomes of women with BC treated with AIs during their postmenopausal period is needed to determine the safest and most effective treatment options for vaginal atrophy. Thus, in this study, we compared the composition of the vaginal microbiomes between BC patients treated with AIs with and without vulvovaginal symptoms. We also hypothesized that despite a similar estrogen status, the microbiomes may differ between these two groups.

## Methods

This study was designed as an analytical cross-sectional study and was performed at a university hospital in Bangkok, Thailand, between March 2022 and November 2022. Postmenopausal BCS aged 50–80 years, treated with letrozole or anastrozole for longer than 6 months, were included. Menopause was defined as no menstruation in the past 12 months, without a hysterectomy. Exclusion criteria included those who had experienced abnormal vaginal bleeding, a history of menopausal hormone therapy or antibiotic use, vaginal douching, vaginal procedures or the use of vaginal products within 7 days, and a history of sexual activity within the previous 48 h. Women who refused to participate or had incomplete data were also excluded. The recruitment process started in March 2022 at the Breast Clinic using the direct approach targeting consecutive eligible patients.

The Human Research Ethics Committee of the Faculty of Medicine at Ramathibodi Hospital approved this study (protocol number MURA2021/961) and confirmed that all research was performed in accordance with the Declaration of Helsinki. All women provided written informed consent for participation in the study.

All eligible participants were divided into two groups according to the presence or absence of GSM based on a validated urogenital atrophy questionnaire, a self-report instrument that describes urogenital atrophy symptoms in BCS^24^. The vaginal symptom group was categorized based on subjective reporting of at least one unpleasant symptom of urogenital atrophy. The control group comprised participants who did not report any GSM symptoms. Demographic information and clinical characteristics were collected by a research assistant based on interviews and medical record reviews. Age, body mass index (BMI), parity, number of vaginal deliveries, medical comorbidities, age of menopause, smoking status, alcohol consumption, sexual behavior, history of chemotherapy, history of radiation exposure, history of tamoxifen use, history of herceptin use, and duration of AI use, were assessed. A pelvic examination was completed by the principal investigator (PP) who was unaware of the participant’s group, including evaluation of the vaginal health index (VHI)^25^. Then, specimens for analysis of the vaginal maturation index (VMI)^26^ and the vaginal microbiome were collected according to standard protocols. The VMI was analyzed by a blinded cytologist. The VHI and vaginal microbiome were evaluated by blinded assessors.

Initially, a pilot study was performed to assess the feasibility of the research and enable calculation of the appropriate sample size. Ten participants were included in the pilot study, five of whom had vaginal dryness, while the other five did not. The results of the pilot microbiome study showed that genus *Sneathia* was prevalent in the group with vaginal dryness but not in the group without this symptom (proportion 0.4 vs 0). The two independent proportions formula was used to calculate the sample size based on the proportion of *Sneathia* in the vaginal microbiome of BCS using AIs who did or did not have symptoms of vaginal dryness. The sample size was predicted as 20 participants per group and thus, 40 participants were recruited.

The primary outcome was the difference in the proportion of genus *Sneathia* in BCS being treated with AIs who did or did not have symptoms of vaginal dryness. *Sneathia* is a genus of Gram-negative, rod-shaped, anaerobic, non-motile bacteria, which has been recently identified as an important contributor to the pathogenesis of gynecological diseases^13^. The secondary outcome was the composition of the vaginal microbiome in each group.

### Collection of vaginal swab samples

The collection of vaginal swab samples and pelvic examination were performed prior to any vaginal procedure. A sterile speculum was used to collect each sample. A disposable vaginal brush was used to collect vaginal discharge from the upper vagina. The samples were placed into a sterile tube filled with DNA/RNA Shield™ reagent (Zymo Research, Irvine, CA, USA).

### DNA extraction

The process of extracting DNA and performing 16S ribosomal RNA gene sequencing was executed by Zymo Research Service (Zymo Research). The DNA extraction procedure involved the utilization of the ZymoBIOMICS® DNA Miniprep Kit (Zymo Research), which was performed following the manufacturer's instructions. The subsequent library preparation step was carried out using the Quick-16S™ NGS Library Prep Kit (Zymo Research) with primers that selectively amplified the V3-V4 region of the 16S ribosomal RNA gene. The final pooled library was subjected to a comprehensive quantification process using TapeStation® (Agilent Technologies, Santa Clara, CA, USA) and Qubit® (Thermo Fisher Scientific, Waltham, WA, USA) to ensure optimal data accuracy and quality. The DNA sequencing was executed by Illumina® MiSeq™ (Illumina, San Diego, CA) with a v3 reagent kit that was spiked with 10% PhiX to optimize the quality of the data generated.

FastQC was used to qualify raw reads. Each individual amplicon sequence variant was analyzed, and the DADA2 pipeline was used to remove errors and chimeric sequences^27^. Qiime v.1.9.1 was used to assign taxonomy and determine alpha and beta diversities^28^. Microbial similarities were analyzed by principle coordinate analysis (PCoA) based on the implementation of Bray–Curtis dissimilarity using the Vegan v2.5-3 package in the R software. Finally, if differences in taxonomic abundance were considered significant between groups, linear determinant analysis effect size (LEfSe) was performed^29^.

### DNA sequencing and statistical analyses

If data were normally distributed, continuous variables were expressed as the mean ± standard deviation, otherwise they were expressed as the median and interquartile range. Chi-square or Fisher’s exact test were performed to appropriately compare various clinical outcomes. The STATA 17.0 program was used for data analysis. A p-value of less than 0.05 was considered statistically significant.

QIIME was used to evaluate alpha diversity, including the Observed species, Chao1 index, Shannon index, and Simpson index. Statistical differences in alpha diversity were evaluated using the Wilcoxon rank-sum test. Beta diversity was evaluated using the Bray–Curtis dissimilarities, weighted Uni-Frac and unweighted Uni-Frac distances.

## Results

### General characteristics

**Figure 1:**
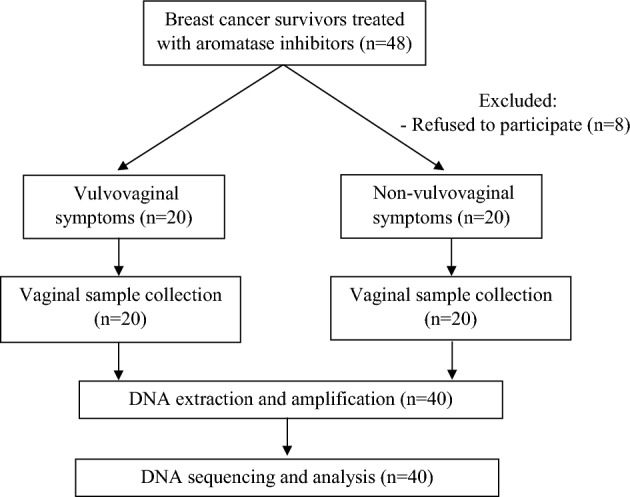
Study flow diagram.

In our study, 48 women were enrolled but eight were excluded because of their refusal to participate. The remaining 40 participants were divided into the vaginal symptom group (n = 20) and the control group (n = 20) based on the urogenital atrophy questionnaire (Fig. 1). Participants’ demographic data are presented in Table 1 by cohort. The characteristics of participants did not differ significantly between the groups, including age, BMI, parity, diabetes, duration of menopause, and clinical details of BC. The majority of the cohort were not sexually active (82.5%) with a mean age of 65 ± 7.5 years and a BMI of 24.5 ± 4 kg/m^2^. The median duration of menopause was 16 years. BC data are depicted in Table 2. The median duration of AI use was 2.6 years. Among BCS, 57.5% (n = 23) received chemotherapy.

**Table 1:** Participants’ demographic data (n = 40).

Characteristics	Vaginal symptom group (n = 20)	Control group (n = 20)	*p*-value
Age^a^ (year); mean ± SD	63.2 ± 5.5	66.8 ± 8.9	0.1408
BMI^a^ (kg/m^2^); mean ± SD	24.1 ± 3.6	24.8 ± 4.4	0.5616
Para^b^; number (%)			0.158
0	7 (35)	10 (50)	
≥ 1	13 (65)	10 (50)	
Number of vaginal deliveries^b^; number (%)			0.172
0	11 (55)	14 (70)	
≥ 1	9 (45)	6 (30)	
Cigarette smoking^b^; number (%)	0 (0)	0 (0)	
Alcohol consumption^b^; number (%)	0 (0)	2 (10)	0.487
Underlying disease^b^; number (%)			
Diabetes mellitus	5 (25)	1 (5)	0.182
Hypertension	6 (30)	8 (40)	0.507
Age at menopause^a^ (year); mean ± SD	49 ± 4.9	48.5 ± 4.4	0.7340
Duration of menopause^c^ (year); mean (IQR)	13.5 (7.5,17.5)	19.5 (8.5,26)	0.1981
History of sexual activity^b^; number (%)	17 (85)	15 (75)	0.695
Sexually active^b^; number (%)	6 (30)	1 (5)	0.091

IQR, Interquartile range.

^a^T-test.

^b^Fischer extraction or Chi-square extraction.

^c^Wilcoxson rank sum test.

**Table 2:** Breast cancer diagnosis and treatment data.

Characteristics	Vaginal symptom group (n = 20)	Control group (n = 20)	*p*-value
Age at breast cancer diagnosis^a^ (year); mean ± SD	59.3 ± 6.4	62.6 ± 9.7	0.2131
History of chemotherapy^b^; number (%)	11 (55)	12 (60)	0.749
History of radiation exposure^b^; number (%)	12 (60)	12 (60)	1.000
History of tamoxifen use^b^; number (%)	4 (20)	3 (15)	1.000
History of Herceptin use^b^; number (%)	3 (15)	2 (10)	1.000
Duration of AIs use^c^ (month); mean (IQR)	35.5 (19, 42)	28 (17, 54.5)	0.7454

AI, aromatase inhibitor; IQR, Interquartile range.

^a^T-test.

^b^Fischer extraction or Chi-square extraction.

^c^Wilcoxson rank sum test.

Regarding the estrogenic status of the vagina, the VMI and VHI, including vaginal pH, were equivalent between the two groups (Table 3). The VMI and VHI findings indicated that the majority of the cohort had a hypoestrogenic status of the vagina with mean scores of 23.4 ± 10.3 and 11.4 ± 2.5, respectively. The mean number of parabasal cells was also comparable between the groups (93.0% vs 92.5%; *P* = 0.9421). No significant differences were observed using the GSM assessment tool (total score: 19.1 vs 20.1; *P* = 0.3314; Table 4).

**Table 3:** Symptoms and signs of vulvovaginal atrophy (mean ± SD).

Characteristics	Vaginal symptom group	Control group	*p*-value
	(n = 20)	(n = 20)	
UAQ score	15.5 ± 1.9	12.8 ± 1.1	0.0000
Irritation from toilet tissue	1.3 ± 0.6	1.2 ± 0.4	0.3781
Irritation from clothing	1.4 ± 0.6	1.1 ± 0.3	0.0999
External genital tenderness	1.1 ± 0.3	1.1 ± 0.3	1.0000
External genital odor	1.2 ± 0.5	1.2 ± 0.4	1.0000
External genital itching	1.3 ± 0.6	1.2 ± 0.4	0.5027
External genital swelling	1.1 ± 0.3	1.0 ± 0.0	0.1544
Vaginal bleeding	1.0 ± 0.0	1.0 ± 0.0	–
Vaginal dryness	3.1 ± 0.2	1.0 ± 0.0	0.0000
Vaginal itching	1.1 ± 0.2	1.0 ± 0.0	0.3236
Vaginal odor	1.2 ± 0.5	1.0 ± 0.0	0.1785
White/creamy discharge	1.0 ± 0.0	1.2 ± 0.4	0.0749
Yellow/greenish discharge	1.0 ± 0.0	1.0 ± 0.0	–
Vaginal health index (VHI)	11.7 ± 2.5	11.1 ± 2.6	0.4196
Elasticity	2.8 ± 0.6	2.5 ± 0.8	0.1606
Fluid volume	2.4 ± 0.6	2.6 ± 0.7	0.3298
pH	1.2 ± 0.4	1.1 ± 0.3	0.6429
Epithelial integrity	2.7 ± 1.0	2.3 ± 0.7	0.2109
Moisture	2.8 ± 0.6	2.6 ± 0.8	0.5013
Vaginal maturation index (VMI)	23.2 ± 10.7	23.6 ± 10.1	0.9042
Parabasal	93 ± 22.5	92.5 ± 20.7	0.9421
Intermediate	0 (0,0)	0 (0,0)	0.9691
Superficial	0 (0,0)	0 (0,0)	0.5736
Vaginal pH	7.5 ± 1.0	7.4 ± 0.9	0.8716

T-test.

**Table 4:** Genitourinary Syndrome of Menopause (GSM) assessment tools (mean ± SD).

Characteristics	Vaginal symptom group (n = 20)	Control group (n = 20)	*p*-value
Elasticity	1.6 ± 0.5	1.7 ± 0.6	0.5602
Lubrication	1.9 ± 0.5	1.8 ± 0.6	0.5472
Tissue integrity	1.8 ± 0.6	2.0 ± 0.6	0.1966
Introitus	1.7 ± 0.6	1.8 ± 0.6	0.5975
Labia majora, minora	1.9 ± 0.5	2.1 ± 0.6	0.1736
Urethra	1.8 ± 0.4	1.9 ± 0.6	0.3500
Rugae	2.1 ± 0.6	2.2 ± 0.6	0.4418
Color	1.7 ± 0.5	1.8 ± 0.6	0.5472
pH	2.0 ± 0	2.0 ± 0	–
Maturation index	2.9 ± 0.7	2.9 ± 0.5	0.7830
Total score	19.1 ± 2.9	20.1 ± 3.8	0.3314

T-test.

### Vaginal microbiome

**Figure 2:**
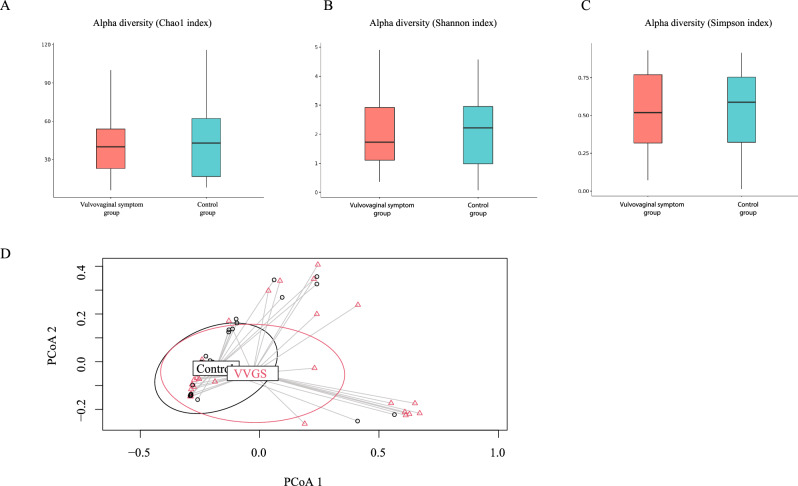
Boxplot showing alpha diversity of vaginal microbiome in breast cancer survivors with and without vaginal dryness by (**A**) Chao1 (richness), (**B**) Shannon index (microbial diversity) and (**C**) Simpson index indicating no significant difference between the groups. (**D**) PCoA generated using Bray–Curtis distance. For each experimental group, an ellipse around the centroid is depicted with no significant separation between the two groups. The control group data were presented as black circles, while the vulvovaginal symptoms group was represented by red triangles. VVGS stands for vulvovaginal symptom.

To investigate whether there was any association between vaginal microbiome and the clinical presentation of patients with BCS receiving or not receiving AIs, several diversity parameters were conducted. Alpha diversity analysis showed no significant difference in vaginal microbial richness between the two groups based on the Observed species, Chao1 index, Shannon index, and Simpson index (Fig. 2A,B). Beta diversity analysis of the vaginal microbiota demonstrated no significant separation by PCoA based on the Bray–Curtis distance and Jaccard index (Fig. 2C,D).

**Figure 3:**
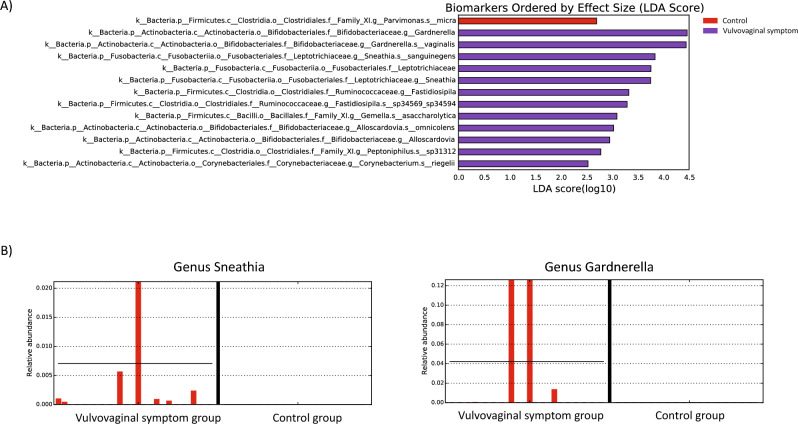
Linear discriminant analysis effect size (LEfSe) analysis of microbial abundance between breast cancer survivors treated with aromatase inhibitors with and without vulvovaginal symptoms. (**A**) Taxa with a significant difference in both groups were detected by LEfSe analysis with a linear discriminant analysis (LDA) (**B**) Bar plot representing relative abundance of *Sneathia* and *Gardnerella* from both groups.

The most common bacterial species observed in this study, based on the average relative abundance across samples, were *Pseudomonas* (100%), *Peptoniphilus* (72.5%), *Varibaculum* and *Streptococcus* (55%) and *Fusobacterium* (52.5%). *Pseudomonas aeruginosa* was dominant in both groups with no significant difference in abundance between groups (Fig. 3). *Lactobacillus* was not dominant in any participant group, and was only detected in two samples (5%), one sample in the vaginal symptom group and one sample in the control group. Analysis of the abundance of differential taxa using Linear discriminant analysis Effect Size demonstrated the significantly elevated abundance of several vaginal microbial taxa in the vaginal symptom group, including *Gardnerella*, *Sneathia*, *Fastidiosipila*, *Gemella*, *Alloscardovia*, *Peptoniphilus* and *Corynebacterium*. An increase in the abundance of *Parvimonas* was identified in the control group (Fig. 3A).

Comparison of the relative abundance showed that *Sneathia sanguinegens* was present in 25% of the vaginal symptom group (5/20), but was not present in the control group. Another bacterial species, *Gardnerella vaginalis*, showed a significant difference in relative abundance between the vaginal symptom group (20%, 4/20) and the control group (0/20). The presence of *Fastidiosipila sp34569-sp34594*, *Gemella asaccharolytica,* and *Alloscardovia omnicolens* was also noted in 20% of the vaginal symptom group (4/20), while they were notably absent in the control group. *Peptoniphilus sp31312* was observed in 15% of the vaginal symptom group (3/20), whereas it was absent in the control group. In the case of *Corynebacterium riegelii*, its presence was identified in 20% of the vaginal symptom group (4/20), while being observed in only 5% of the control group (1/20). In contrast, within the control group, there was a notable increase in the prevalence of *Parvimonas micra*, which was present in 25% of the control group, while absent in the vaginal symptom group.

## Discussion

Vulvovaginal symptoms are an undesirable side-effect in BCS treated with AIs that increase the burden of illness^9^. Although VVA usually results from atrophic vaginitis caused by aging and hypoestrogenic changes, this did not completely explain the occurrence of vulvovaginal symptoms in BCS treated with AIs, and some such patients do not report symptoms such as vaginal dryness, itching, discharge and pain during intercourse. It was therefore questionable whether components of the vaginal microbiome elicit the symptoms of VVA. In this study, we analyzed the composition of the vaginal microbiome between BCS treated with AIs with and without vulvovaginal symptoms. This prospective study found that the relative abundance of *Sneathia* and *Gardnerella* was significantly increased in BCS treated with AIs with vulvovaginal symptoms, whereas the relative abundance of *Parvimonas* was increased in patients without vulvovaginal symptoms. However, in this study, we did not detect differences in bacterial diversity and richness in BCS treated with AIs with vulvovaginal symptoms.

Little is known about the composition of the vaginal microbiome in postmenopausal women, its interactions with estrogen, or its associations with vulvovaginal symptoms. The lactobacilli are considered to have a positive effect on genital health via the modulation of host immune responses and the subsequent protection against genital infections^30,31^. The microbial composition of the vaginal microbiome has been categorized into five community state types (CSTs), determined by the relative abundance of four *Lactobacillus* species: *L. crispatus, L. gasseri, L. iners, and L. jensenii*^32^. A distinct bacterial community (CST IV-A) with a low relative abundance of *Lactobacillus* has been linked to a variety of vaginal health problems including VVA^21,22^. The absence of lactobacilli can lead to vaginal microbiome dysbiosis because lactobacilli exert their protective effects through several mechanisms, such as competing for adherence to the vaginal epithelium, producing lactic acid to lower vaginal pH and exert antimicrobial activity against non-resident microbiota, generating hydrogen peroxide to suppress the growth of various microorganisms, and modulating the local immune system^33^. Our discoveries based on 16S gene analysis were not consistent with previous research in healthy postmenopausal women^21,22,34,35^. *Lactobacillus* was not found to be universally present in our study. This result was consistent with the findings of Balmaganbetova et al., who evaluated the vaginal microbiotas of 278 women with various biological subtypes of BC. The authors found a significant decrease in the total number of *Lactobacillus* spp. to below normal levels in patients with all subtypes of BC, indicating vaginal dysbiosis^36^. Our study population was specifically BCS treated with AIs who have a significantly lower serum estrogen level than postmenopausal women in general. As a result of their postmenopausal status, and BC and estrogen deprivation therapy, GSM, especially associated with VVA, should be assessed among BCS to provide timely and appropriate treatment. Based on the potential therapeutic role of probiotic treatments to restore healthy vaginal microbiota by itself and even combined with standard antibiotic treatments, leading to several studies applying different lactobacilli combinations, concentrations, and administration interventions to treat GSM and bacterial vaginosis^37^. A probiotic treatment using an orally administered *Lactobacillus* spp. preparation was studied in a randomized placebo-controlled double-blinded pilot project. Marschalek et al.^38^ reported a positive trend toward improvement in the Nugent score during therapy in postmenopausal patients with BC undergoing chemotherapy. Of interest for future research, is the efficacy and safety of probiotic therapies or new interventions to recover the vaginal microbiome and reduce vulvovaginal symptoms caused by VVA in BCS.

In this study, we hypothesized that the composition of the vaginal microbiome of AI-treated BCS who experienced vaginal symptoms would be different from that of women who did not experience these symptoms. Bioinformatics analysis revealed that there was no difference in the diversity or richness between BCS treated with AIs with vulvovaginal symptoms and those without vulvovaginal symptoms. These results were consistent with previous studies among postmenopausal patients. Shen et al.^39^ reported that the overall diversity and evenness of bacterial communities were not significantly different between healthy women and those with atrophic vaginitis. Whereas, Hummelen et al.^21^ demonstrated that the microbiota abundance profile showed increased bacterial diversity in women experiencing moderate to severe vaginal dryness. This inconsistency may be due to differences in population characteristics. Our study focused on BCS treated with AIs who may have had much lower estrogen levels than postmenopausal women in general^5,6^. Future studies based on a larger population may lead to a better understanding of the vaginal microbiome composition during different vaginal states/diseases.

Most previous studies correlating the vaginal microbiome with genitourinary atrophy have relied primarily on the prevalence of *Lactobacillus* species^21–23^. In the absence of *Lactobacillus*, we found the relative abundance of *S. sanguinegens* and *G. vaginalis* to be significantly different in the vaginas of BCS treated with AIs with vulvovaginal symptoms compared with that in the vaginas of those without vulvovaginal symptoms.

*Sneathia* is a genus of Gram-negative, rod-shaped, anaerobic, non-motile bacteria. Taxonomically, *Sneathia* belongs to the family *Leptotrichiaceae*^40^. Recently, *Sneathia* infections have been reported to be associated with important gynecologic diseases or conditions^13,41–43^. In vaginal infection, *S. sanguinegens* is capable of inducing the secretion of inflammatory cytokines, including IL-1α, IL-1β and IL-8 from human vaginal epithelial cells in vitro^44^. It also has the potential role as a secondary colonizer in vaginal biofilms^16^. In one association study, an increased relative abundance of *S. sanguinegens* in the vaginal microbiome was associated with the presence of clue cells and bacterial biofilms in vaginal fluids, suggesting that *Sneathia* spp. may be important contributors to the process of inflammation in vagina^45^. However, some previous studies did not support the hypothesis of the linkage between *Sneathia* and vaginal health. Hummelen et al.^21^ demonstrated that there was a low abundance (< 1%) of *Sneathia* in a study set of postmenopausal women. This may be explained by the small sample size used in the study or the fact that the study assessed the vaginal microbiome in postmenopausal women without accounting for estrogen status. Shen et al.^39^ also reported that *Sneathia* was not associated with the clinical symptoms of atrophic vaginitis in postmenopausal women. A comprehensive understanding of this association is warranted for the development of effective prophylactic and therapeutic approaches for vulvovaginal symptoms in the future.

*G. vaginalis* is a Gram-negative opportunistic pathogen that may be the cause of some of the clinical features of bacterial vaginosis^46^. Shen et al.^39^ demonstrated that *Gardnerella* was significantly more abundant than *Lactobacillus* in the vaginal communities of women with atrophic vaginitis. Conversely, Mitchell et al. and Hummelen et al. did not reveal any correlation between reported menopausal genitourinary symptoms and a vaginal microbiota including *G. vaginalis*^21,23^. In our study, *G. vaginalis* was evidently prevalent in the vaginas of BCS treated with AIs who experienced vulvovaginal symptoms. The possible mechanism may involve the putative virulence determinants of *G. vaginalis* that include mucin degradation, cytotoxicity, hemolysis, adhesion to the epithelium, biofilm production, iron scavenging and antimicrobial resistance^47,48^. Furthermore, *Gardnerella* has a propensity to produce a member of the cholesterol-dependent family of pore-forming toxins (vaginolysin), which are leading to vaginal epithelial cells lysis and death^49,50^. Nonetheless, the association between *G. vaginalis* and vulvovaginal symptoms remains to be fully elucidated. Future research is needed to determine how the vaginal microbiome and/or individual bacterial species in the vagina influence vaginal health and GSM symptoms.

For another genus that increased in prevalence within the vaginal symptom group, they were also found to be associated with particular gynecologic diseases. For instance, *Fastidiosipila*, a Gram-positive, coccus-shaped organism, was found to be a biomarker for gynecologic diseases such as hrHPV infection in pregnant women^51^. Conversely, some studies noted an increased abundance of *Fastidiosipila* in the control group when compared to the adenomyosis or genital lichen sclerosus group^52,53^. *Gemella* was significantly enriched in patients with *Trichomonas vaginalis* infection and was associated with a higher risk of HIV-1 acquisition^54,55^. *Alloscardovia* has been identified as being associated with an inflammasome-dependent immune network and is usually regarded as a bacterium responsible for atrophic vaginitis^56^.

Concerning the clinical application, menopausal hormone therapy is currently not recommended for women with a history of BC. This is due to the fact that there is possible recurrence of disease, in which there are no standard options of treatment proven to be effective at this time^9,10^. Therefore, this leads to further steps in discovering more novel therapies aimed for individualized patients. Probiotics have been shown to be effective therapy to restore vaginal homeostasis and also to avoid future pathogen adhesion or colonization in BCS experiencing bothersome vaginal symptoms^38,57^. Further developments of newer therapies should benefit this group of patients in the future.

This study has several strengths. First, because of a lack of previous research in this specific area, we performed a pilot study included 10 women to assess the feasibility of the research and to calculate the appropriate sample size. Second, although previous studies had focused on the vaginal microbiome in patients with and without genitourinary symptoms, no previous study had evaluated the vaginal microbiome in AI-treated BCS. In addition, we used the validated urogenital atrophy questionnaire, a self-report instrument that describes urogenital atrophy symptoms and was designed for BCS to divide patients into two groups. This instrument is reliable and valid regardless of the participant’s level of sexual activity, partner status or sexual orientation^21^. We also evaluated signs of vaginal atrophy including the VMI and VHI vaginal to address the vaginal hypoestrogenic status. Finally, the DNA extraction kits, the use of appropriate controls and the reference database that was used as the standard at the time of the study resulted in no exclusion of participants as a result of poor DNA quality.

Our study has some limitations. First, a small number of patients was included in the analysis and our findings need to be verified by a larger patient cohort in the future. Second, the lack of confirmation of serum estrogen levels may have led to some patients not having their hypoestrogenic status verified. Third, this is a cross-sectional study in which the cause-and-effect is difficult to determine between vulvovaginal symptoms and the vaginal microbiome. Finally, this study only evaluated AI-treated BCS; therefore, our findings have limited generalizability. To fully elucidate the mechanisms involved, further studies involving larger sample sizes, quantification of different *Lactobacillus* and primary/opportunistic pathogens, and different populations are needed.

## Conclusion

To our knowledge, this study is the first to explore the vaginal microbiomes of AI-treated BCS. We found that *Sneathia* and *Gardnerella* were present in higher relative abundance in the vaginas of women who reported vaginal symptoms compared with those in the vaginas of women who reported no such symptoms. There was no difference in the diversity or richness of the vaginal microbiome between the groups. We also highlight that in the absence of *Lactobacillus*, other bacteria may play a role in the genitourinary symptoms of menopause, especially vaginal symptoms.

## Data Availability

The data presented in this study are available on request from the corresponding author. The data are not publicly available because of the ethics and rights of the Faculty of Medicine, Ramathibodi Hospital, Mahidol University. The DNA sequence used in this study was available in the Genbank repository (BioProject PRJNA999136; Reviewer’s link is https://dataview.ncbi.nlm.nih.gov/object/PRJNA966136?reviewer=bj8f7kr1j19qcmbts9g5s4vd8).
